# Das medizinische Atom: Radioisotope in internistischen Kliniken der Bundesrepublik Deutschland, 1945 bis 1965

**DOI:** 10.1007/s00048-025-00415-7

**Published:** 2025-05-13

**Authors:** Ursula Heim

**Affiliations:** Institut für Ethik, Geschichte und Theorie der Medizin, Universität Münster, Von-Esmarch-Straße 62, 48149 Münster, Deutschland

**Keywords:** Radioisotope, Innere Medizin, Medizinische Grundlagenforschung, Klinische Forschung, Radioisotopes, Internal Medicine, Basic Research, Clinical Research

## Abstract

Radioisotope zählten zu den prägenden Neuerungen der klinisch-internistischen Medizin der Nachkriegszeit. Medizinische Universitätskliniken setzten Radioisotope in der Therapie bislang nicht behandelbarer Krankheiten ein und nutzten sie gleichzeitig zur Untersuchung (patho-)physiologischer Prozesse im Labor. Mitte der 1950er Jahre wurde mit neuen technischen Methoden – wie der Szintigrafie – das diagnostische und therapeutische Repertoire der Inneren Medizin erweitert. Der Beitrag arbeitet am Beispiel der Isotopenforschung die Bedeutung technikgetriebener Wandlungsprozesse für die internistische Klinik der frühen Bundesrepublik heraus. Darüber hinaus werden Transferprozesse zwischen medizinischer Grundlagenforschung und klinischer Forschung in den Blick genommen und als eine Form der Translationalen Medizin *avant la lettre* analysiert.

## Abstract (en)

Radioisotopes were one of the defining innovations in clinical internal medicine in the post-war period. Medical university clinics used radioisotopes in the treatment of previously untreatable diseases and at the same time used them to study physiological processes in the laboratory. In the mid-1950s, new technical methods—such as scintigraphy—expanded the diagnostic and therapeutic repertoire of internal medicine. The article uses the example of isotope research to highlight the significance of technology-driven processes of change for the internal medicine clinic in West Germany. In addition, transfer processes between basic medical research and clinical research are examined and analyzed as a form of translational medicine avant la lettre.

Zu den prägendsten Neuerungen der klinischen Medizin in der Nachkriegszeit gehörte die Implementierung radioaktiver Isotope in Grundlagenforschung, Diagnostik und Therapie. Kernchemische Techniken ermöglichten das Studium physiologischer Prozesse im Organismus, die vorher einer detaillierten Erforschung nicht zugänglich waren. Der Berner Klinikdirektor Walther Frey (1884–1972) betonte als Vorsitzender der Deutschen Gesellschaft für Innere Medizin in seiner 1950 gehaltenen Kongressrede: „Die Isotopenforschung ist völliges Neuland. Man ist von der molekularen Chemie zur Atomphysik übergegangen; eine Wendung von größter Bedeutung, auch für die Medizin, in diagnostischer, vielleicht auch therapeutischer Hinsicht.“ (Frey 1950: 2) Freys Einschätzung war erkennbar beeinflusst von der Aufbruchstimmung, die die deutschsprachige Medizin der Nachkriegszeit ergriffen hatte. Gleichwohl war seine Beobachtung gut begründet. An zahlreichen internistischen Kliniken hatte man begonnen, sich mit radioaktiven Isotopen zu befassen; erste Forschungsergebnisse wurden in medizinischen Fachzeitschriften wie der *Deutschen Medizinischen Wochenschrift* und der *Klinischen Wochenschrift* veröffentlicht (z. B. Boll et al. 1951; Odenthal 1951; Goldeck et al. 1952). Nur ein Jahrzehnt später ergab sich ein deutlich anderes Bild: Diagnostische und therapeutische Verfahren mit Radioisotopen hatten sich breit etabliert und waren fester Bestandteil der internistischen Klinik geworden. „Radioisotope“, so konstatierte 1960 der Wiener Internist und Pionier der Nuklearmedizin Herbert Vetter (1920–2009),

„werden in zahlreichen Gebieten der klinischen Forschungsarbeit verwendet. Die physiologischen Prozesse von Gesundheit und Krankheit werden untersucht; die Resultate verschiedener Behandlungsformen können überprüft werden; in die praktischen Alltagsprobleme, die bei der Behandlung der Patienten auftreten, versucht man so Licht zu bringen. Heute gibt es in der Medizin kein einziges Fach mehr, in das Isotope nicht Eingang gefunden hätten“ (Vetter 1960: VIII).

Hier setzt dieser Beitrag an. Der Untersuchungszeitraum von 1945 bis 1965 konzentriert sich auf die Phase, in der Internist:innen radioaktive Isotope als Teil technikgetriebener Wandlungsprozesse in der Klinik implementierten. Die Innere Medizin, eine der beiden Ursprungsdisziplinen der Nuklearmedizin in der Bundesrepublik, bietet daher ein spannendes Untersuchungsfeld: Einerseits kamen aus internistischen Kliniken entscheidende Impulse für die Einführung kernchemischer Pharmaka und Techniken in Diagnostik und Therapie, wie am Beispiel der Medizinischen Klinik Wien aufgezeigt wurde (Bayer 2022). Andererseits verstand sich die Innere Medizin bis ins späte 20. Jahrhundert als „das zentrale klinische Grundlagenfach“ (Buchborn 1980: 121) und beanspruchte, Repräsentantin der klinischen Medizin zu sein.

Der Beitrag ist als Fallstudie angelegt und geht aus einem Projekt zur Wissenschaftsgeschichte der klinischen Medizin in der Nachkriegszeit und frühen Bundesrepublik hervor.^1^ Anhand systematischer Analysen der Verbreitung und Nutzung von Radioisotopen wird die enge Verbindung zwischen Grundlagenforschung und klinischer Forschung in der Inneren Medizin untersucht. Hierbei liegt der Fokus auf den Besonderheiten der kliniknahen Grundlagenforschung als Teil der Medizinischen Klinik, die durch den Einsatz von Radioisotopen neue Forschungsansätze ermöglichte. Die interdisziplinäre Zusammenarbeit zwischen Innerer Medizin, Radiologie und Medizinischer Physik spielte dabei eine zentrale Rolle: Sie eröffnete nicht nur neue technische Möglichkeiten in Diagnostik und Therapie, sondern führte auch zu einem Wandel der Forschungskultur innerhalb der klinischen Medizin.

Bisherige Arbeiten beleuchteten die Bedeutung der Radioisotope in verschiedenen Kontexten: zum einen im Rahmen der Wissenschaftsgeschichte der Molekularbiologie (Rheinberger 2007), zum anderen in Bezug auf die Herausbildung der Nuklearmedizin als eigenständiger Disziplin (Feld & De Roo 2000; Winkler 1989). Bettina Hitzer thematisiert in ihrer umfassenden Studie zur Geschichte der Krebserkrankung exemplarisch den Einsatz strahlentherapeutischer Großgeräte in der klinischen Medizin der Nachkriegszeit (Hitzer 2020). Mit förderpolitischen Aspekten der Strahlenforschung in der Bundesrepublik befasst sich Alexander von Schwerin anhand des Schwerpunktprogramms Radiologie der DFG (von Schwerin 2015). Die Forschung an und mit Radioisotopen in der DDR wurde bereits überblicksartig thematisiert (Deckart et al. 1999; Gausemeier 2020). Sybille Marti untersucht detailliert die Entwicklung der Kerntechnologie in der Schweiz. Dabei stehen medizinische Anwendungen im Spannungsfeld zwischen militärischen Interessen der biologischen Strahlenforschung und der zivilen Nutzung radioaktiver Isotope im klinischen Einsatz (Marti 2021). Florian Bayer analysiert die Etablierung radioaktiver Isotope an der Universitätsklinik Wien vor dem Hintergrund der internationalen Atompolitik des Kalten Krieges (Bayer 2022). Studien zur US-amerikanischen Isotopenforschung von Angela Creager (2013) sowie zum britischen Isotopenprogramm von Alison Kraft (2006) legen den Schwerpunkt auf Zusammenhänge zwischen (forschungs-)politischen Entwicklungen und technologischen Errungenschaften.

Die Bedeutungselemente und die Entwicklung des Konzepts „Grundlagenforschung“ wurden verschiedentlich in der wissenschaftshistorischen Forschung untersucht: Die Wandlungen des Wissenschaftsverständnisses in den naturwissenschaftlichen Disziplinen zeigt Désirée Schauz in einer umfassenden begriffshistorischen Untersuchung auf (Schauz 2020). Gregor Lax arbeitet den Bedeutungszuwachs des Konzepts „Grundlagenforschung“ in der Nachkriegszeit anhand forschungspolitischer Diskurse heraus. In der Hierarchiebildung zwischen Grundlagenforschung und Anwendungsforschung wurde die freie, reine Grundlagenforschung in der Nachkriegszeit als spezifisches Kulturgut der Demokratie deklariert (Lax 2015: 208). Während Lax den Bedeutungszuwachs der Grundlagenforschung betont, fokussiert sich Carola Sachse auf deren organisationsspezifische Entwicklung in der Max-Planck-Gesellschaft (MPG). Die MPG positionierte sich „nach dem Krieg als exklusive Institution der bundesdeutschen Grundlagenforschung“ (Sachse 2015: 255). Martina Schlünder untersucht die medizinische Forschung in der MPG als „heterogenes Feld von Forschungsinhalten und -methoden, angesiedelt zwischen biologischer Grundlagenforschung und klinischer Praxis“ (Schlünder 2024: 398). Insgesamt zeigt sie, dass die medizinische Forschung in der Bundesrepublik mit erheblichen Herausforderungen – insbesondere im Bereich der klinischen Forschung – konfrontiert war. Bemühungen der MPG, klinische Forschung, beispielsweise durch die Implementierung von Forschungskliniken in den 1950er und 1960er Jahren, zu stärken, scheiterten. Ferner konstatiert sie für die medizinischen Forschungsinstitute der MPG eine Hinwendung zur „molekularbiologischen und neurowissenschaftlichen Grundlagenforschung“ (ebd.: 401), die sich von der klinischen Medizin stark entfernte.

Anders stellte sich die Situation in den Medizinischen Universitätskliniken dar: Auch hier wurde in den 1950er Jahren das Verhältnis von theoretischer (grundlagenorientierter) und klinischer Forschung akut, jedoch in spezifischer Weise diskutiert. Forschung zu Mechanismen und Prozessen der Krankheitsentstehung war seit Langem ein fester Bestandteil der Forschung in Kliniken und ging unmittelbar aus klinischen Beobachtungen hervor, wie der Heidelberger Internist Karl Matthes (1905–1962) prägnant zusammenfasste:

„Der Auftrag des Patienten an den Arzt kennt somit keine Grenzen, durch die etwa die Aufgaben der Klinik von den theoretischen Instituten getrennt werden könnten. Entsprechend kann sich eine, von einer klinischen Fragestellung ausgehende wissenschaftliche Arbeit, bei vorhandenen methodischen Möglichkeiten […], auf Grund der ihr innewohnenden eigenen Dynamik bis tief in die Probleme der Grundlagenforschung entwickeln“ (Matthes 1962: 6).

Grundlagenforschung in der Inneren Medizin orientierte sich somit eng an klinischen Erfordernissen. Experimentelle Untersuchungen zur Klärung pathophysiologischer und pathogenetischer Fragestellungen fanden sowohl direkt an Patient:innen in der Klinik als auch in speziell eingerichteten Laboratorien statt. Diese Labore, ausgestattet für Tierexperimente und In-vitro-Forschungen, boten in Medizinischen Kliniken die notwendige Infrastruktur dafür. Technologische Errungenschaften ermöglichten nicht nur die Einrichtung hochwertiger und leistungsfähiger Labore, sondern erweiterten das diagnostische und therapeutische Handlungsspektrum erheblich. Dabei rückten die technische Machbarkeit, Genauigkeit und Validität diagnostischer Techniken sowie die Wirksamkeit und Sicherheit therapeutischer Interventionen als zentrale Bestandteile der klinischen Forschung in den Fokus.

Vor diesem Hintergrund adressiert der Beitrag drei zentrale Forschungsfragen: 1.) Wie verlief die Implementierung von Radioisotopen in der Inneren Medizin während der Nachkriegszeit und frühen Bundesrepublik? 2.) In welcher Weise erweiterten kernchemische Pharmaka und Techniken das diagnostisch-therapeutische Handlungsspektrum in internistischen Kliniken? 3.) Was zeigt das Beispiel der Isotopenforschung für das Verhältnis von Grundlagenforschung und klinischer Forschung in der Inneren Medizin? Ausgehend von diesen Fragestellungen untersucht der vorliegende Beitrag die Forschung an und mit Radioisotopen in vier Schritten: Zunächst wird im ersten Teil durch eine quantitative Analyse einschlägiger medizinischer Fachzeitschriften die Implementierung radioaktiver Isotope in der Inneren Medizin beleuchtet. Daran anschließend erfolgt eine qualitative Untersuchung ausgewählter Veröffentlichungen: Die Analyse nimmt zunächst den frühen diagnostischen und therapeutischen Einsatz radioaktiver Isotope in den Blick. Im Rahmen technikgetriebener Wandlungsprozesse der Inneren Medizin wird die Diagnostik mit Szintigrafen untersucht. Die qualitative Analyse endet mit ausgewählten Beispielen zur Forschung mit der Indikatormethode in internistischen Kliniken. Abschließend werden im vierten Teil die gewonnenen Erkenntnisse diskutiert. Als Quellen der systematischen Analyse dienten drei für die deutschsprachige Innere Medizin besonders relevante Fachzeitschriften, ausgewählte Lehrbücher und Tagungsbände. Die Auswahl der Fachzeitschriften basierte darauf, dass sie in ihrer Zeit maßgeblich für die Diskussion und Verbreitung von Forschungsergebnissen und neuen medizinischen Praktiken waren. Zudem gaben sie einen generellen Überblick über die Implementierung neuer Methoden – wie den Einsatz von Radioisotopen – in internistischen Kliniken. Lehrbücher wurden berücksichtigt, da sie den allgemeinen Kenntnisstand der Inneren Medizin in systematischer Weise zusammenfassten und ermöglichten, die Bedeutung von Radioisotopen im größeren Kontext der medizinischen Praxis zu verstehen. Tagungsbände boten darüber hinaus Einblicke in fachinterne Diskussionen zur Etablierung radioaktiver Isotope in die Medizin. Technische Instrumente wie Szintigrafen und Geiger-Müller-Zählrohre werden zudem näher betrachtet, da dies für das Verständnis der Implementierung von radioaktiven Isotopen in der Inneren Medizin notwendig ist.

Zunächst wird nun der historische Hintergrund beleuchtet, der die Einführung von Radioisotopen in die Medizin ermöglichte. Daran anschließend kehre ich zur Inneren Medizin zurück und lege offen, wie sich Radioisotope in internistischen Kliniken der Bundesrepublik verbreitet haben.

## Der Weg der Radioisotope in die Medizin

Die Entdeckung der künstlichen Radioaktivität durch Irène Joliot-Curie (1897–1956) und Frédéric Joliot-Curie (1900–1958) ebnete der Etablierung von künstlichen radioaktiven Isotopen^2^ in der medizinischen Forschung den Weg. Die frühe Forschung zu Radioisotopen durchlief verschiedene Phasen und wurde von unterschiedlichen Institutionen geprägt: In den 1930er und 1940er Jahren erfolgte die frühe Anwendung der Tracermethode^3^ im Deutschen Reich in enger Verflechtung von Wissenschaft, Industrie und militärischen Interessen (von Schwerin 2009: 27). Georg von Hevesy (1885–1966), der 1926 zunächst eine Professur in Freiburg annahm und 1929 zum Ordinarius für Physikalische Chemie ernannt wurde, führte erste physiologische Untersuchungen mit radioaktiven Isotopen durch. Nach der Machtübernahme durch die Nationalsozialisten emigrierte Hevesy nach Dänemark, wo er die Indikatorforschungen an der Schnittstelle von Chemie und Medizin fortsetzte (Niese 2004). Seine Arbeiten mit P^32^ am Stoffwechsel der Ratte gingen als Pionierarbeit in die Medizingeschichte ein. Während des NS-Regimes unterstützte die Auergesellschaft als bedeutende industrielle Akteurin die Errichtung einer Neutronenanlage am Kaiser-Wilhelm-Institut für Hirnforschung in Berlin-Buch (von Schwerin 2009: 17). Die Forschung mit radioaktiven Isotopen in der Genetischen Abteilung des Instituts lieferte Erkenntnisse für die medizinische Diagnostik (Bielka 1997: 47). In den Anfängen der Radioisotopenforschung spielten Biophysiker:innen und Radiochemiker:innen eine zentrale Rolle bei der Integration der Tracermethode in die biologischen Wissenschaften und die Medizin – sowohl vor als auch während des Zweiten Weltkriegs. Die biologische Strahlenforschung wurde während des Krieges im Kontext militärischer Interessen fortgeführt und durch die Notgemeinschaft der Deutschen Wissenschaft finanziell gefördert.

Die Anwendung von Isotopen in der Klinik begann vor dem Zweiten Weltkrieg. Radioisotope wurden als experimentelle Therapien für bisher nicht behandelbare Krankheiten eingesetzt. John H. Lawrence (1904–1991) behandelte bereits ab 1936 maligne hämatologische Erkrankungen mit Radiophosphor (P^32^). Die Ergebnisse dieser Therapieversuche stellte er 1942 in einer Übersichtsarbeit vor: Mit P^32^ konnten bei myeloischer Leukämie, Polycythaemia vera und beim Lymphosarkom Remissionen erzielt werden (Low-Beer et al. 1942). Die USA hatten mit dieser experimentellen Erprobung radioaktiver Isotope am Menschen international eine Führungsrolle inne. Bereits in der Anfangsphase waren interdisziplinäre Kooperationen, der Ausbau technischer Infrastruktur und medizinische Forschungsinteressen eng verzahnt (Creager 2002: 370).

Die restriktiven Exportbestimmungen der US-amerikanischen Regierung nach dem Zweiten Weltkrieg schränkten bis 1947 die internationale Verfügbarkeit von Radioisotopen erheblich ein (Creager 2009: 222). Die erste nukleare Reaktoranlage Europas in Harwell, Großbritannien ermöglichte die Radioisotopenherstellung auf industriellem Niveau. Das britische Isotopenprogramm hatte weniger strikte Regularien für den internationalen Export von Isotopen, wodurch es für die Verfügbarkeit von Radioisotopen für medizinische Forschungsvorhaben international von entscheidender Bedeutung war (Kraft 2006: 17). Die medizinische Verwendung radioaktiver Isotope wurde ab den späten 1940er Jahren als zivile Nutzung von Kernenergie sowohl forschungspolitisch als auch medial angepriesen. Die Implementierung von Radioisotopen in der Medizin spielte sowohl in der „Atoms for Peace“-Kampagne der US-amerikanischen Regierung als auch auf der ersten internationalen Konferenz zur Atomenergie 1955 in Genf eine zentrale Rolle.

Die Kerntechnologie galt in den 1950er und 1960er Jahren als Zukunftstechnologie, mit der „nicht nur die weltweit geplanten eher profanen Kernkraftwerke zur Energiegewinnung, sondern auch fantastische Zukunftsvisionen von Meerwasserentsalzungen, bewässerten Wüsten, einer begrünten Antarktis bis hin zu technischen Revolutionen in der Medizin und Chemie“ (Raithel & Weise 2022: 68) verknüpft waren. In der „Atomeuphorie der 1950er und 1960er Jahre“ (Radkau 2017: 131) lag der Fokus auf der Nutzung des sogenannten „friedlichen Atoms“. Bis 1955 war die Nutzung der Kernenergie in der Bundesrepublik streng geregelt: Voraussetzung für die Beschaffung von radioaktiven Isotopen war zum einen die Erlaubnis der alliierten Besatzungsbehörden in den westdeutschen Staaten. Durch das Kontrollratsgesetz Nr. 25 waren Forschungsvorhaben in den Bereichen Atomphysik und Kernenergie auch im zivilen Bereich genehmigungspflichtig (von Schwerin 2015: 309). Der Import und die Verteilung von Radioisotopen wurden zum anderen in Absprache mit den britischen Besatzungsbehörden zentral geregelt. In diesem Rahmen waren die Universität Göttingen und die Max-Planck-Gesellschaft in der direkten Nachkriegszeit bedeutsame Akteurinnen des Imports und der Distribution. Am Institut für Medizinische Prüf- und Testmethoden, ab 1947 Medizinische Forschungsanstalt (MFA), war die Infrastruktur für die zentrale Beschaffung und Verteilung von Isotopen vorhanden. Das Isotopenlabor in Göttingen belieferte Kliniken mit importierten Radioisotopen (ebd.: 319). Deutsche Forscher:innen konnten ab 1948 Radioisotope aus Harwell beziehen und in der Klinik einsetzen. In welchem Umfang die importierten Radioisotope als Erweiterung des klinischen Handlungsspektrums in der Inneren Medizin Eingang fanden, zeigt nachfolgend eine quantitative Auswertung relevanter medizinischer Fachzeitschriften.

## Systematische Analyse der Radioisotopenforschung in internistischen Kliniken

Systematisch ausgewertet und in einer Datenbank erfasst wurden Originalbeiträge und Sammelreferate beziehungsweise Übersichten dreier für die Innere Medizin zentraler Zeitschriften: *Deutsche Medizinische Wochenschrift *(1946–1965), *Klinische Wochenschrift *(1946–1965), *Ärztliche Wochenschrift/Der Internist* (1947–1965).^4^ Kurze wissenschaftliche Mitteilungen sind nicht in die quantitative Auswertung eingeflossen. Folgende Einschlusskriterien waren für die Aufnahme eines Fachbeitrags in die Datenbank entscheidend: 1.) Die Mediziner:innen setzten Radioisotope im Labor oder in der Klinik ein. 2.) Der Fachartikel stammte aus einer internistischen Klinik des deutschsprachigen Raums, wobei sowohl Beiträge aus akademischen als auch aus nicht-akademischen Einrichtungen aufgenommen wurden.

Als Kriterien der Differenzierung in der Datenbank sind neben den Namen der Autor:innen, Geschlecht, Jahr, Ort und Land die verwendeten Isotope sowie die Organsysteme der Inneren Medizin erfasst. Des Weiteren wurden in der Datenbank alle Fachbeiträge nach der Ausrichtung des Forschungsvorhabens – Medizinische Grundlagenforschung oder Klinische Forschung – eingeteilt. Die Einteilung der Fachbeiträge unter „Medizinische Grundlagenforschung“ setzt sich zusammen aus Laborexperimenten und Humanexperimenten an Gesunden sowie Patient:innen zur Ermittlung physiologischer Parameter und Generierung pathophysiologischer Erkenntnisse. Unter „Klinische Forschung“ wurden Fachbeiträge eingruppiert, welche die Anwendung von Radioisotopen als diagnostische und therapeutische Verfahren an Patient:innen thematisieren. Insgesamt konnten für den Untersuchungszeitraum 156 Fachbeiträge zur Radioisotopenforschung aus der Inneren Medizin identifiziert werden. Von diesen 156 waren 142 Originalbeiträge und 14 Sammelreferate, die in der Datenbank anhand der eingangs genannten Kriterien erfasst wurden.

Die ermittelten Beiträge werden anhand von vier Kriterien aufgeschlüsselt: 1.) Zunächst wird die Verteilung der Fachbeiträge auf die einzelnen Jahre des Untersuchungszeitraums dargestellt. 2.) Darauf folgt eine Aufteilung der Fachbeiträge nach Ausrichtung des Forschungsvorhabens in medizinische Grundlagenforschung und klinische Forschung. 3.) Eine weitere Differenzierung für die Anwendung von Radioisotopen orientiert sich an den verschiedenen Organsystemen. 4.) Zur Beantwortung der Frage, an welchen Kliniken schwerpunktmäßig Isotopenforschung durchgeführt wurde, ist schließlich der Ort das vierte Kriterium der quantitativen Auswertung.

### Verteilung der Fachbeiträge

**Abb. 1:**
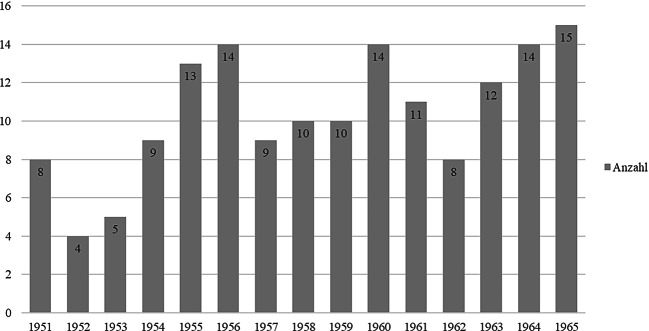
Fachbeiträge zu Radioisotopen aus internistischen Kliniken

Erste Veröffentlichungen von Internist:innen zur Radioisotopenforschung fanden sich ab 1951. Das Diagramm zeigt, dass die Anzahl der Fachbeiträge aus internistischen Kliniken im Verlauf des Untersuchungszeitraums zwar zugenommen, jedoch kein starker Anstieg stattgefunden hat (Abb. 1). Es finden sich von 1951 bis 1965 kontinuierlich Fachbeiträge zu radioaktiven Isotopen aus internistischen Einrichtungen.

### Ausrichtung der Forschungsvorhaben

**Abb. 2:**
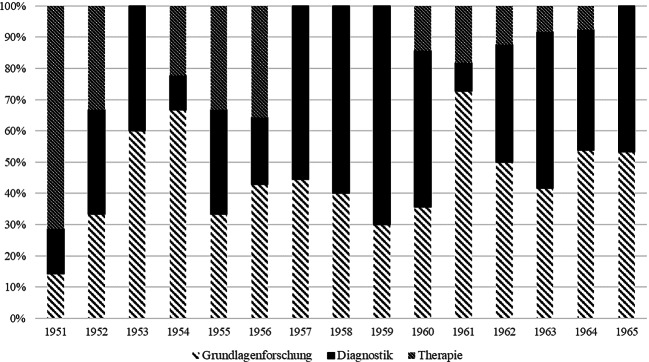
Verteilung internistischer Fachbeiträge nach Ausrichtung des Forschungsvorhabens

Für den Untersuchungszeitraum waren insgesamt 69 Fachbeiträge der Grundlagenforschung und 84 der klinischen Forschung zuzuordnen. Drei Sammelreferate aus den Jahren 1951, 1952 und 1955 fielen in die Rubrik Verschiedenes, da Internist:innen den gesamten zeitgenössischen Kenntnisstand der Isotopenforschung zusammenfassten. Für die klinische Forschung mit Radioisotopen wurde eine weitere Differenzierung der Beiträge in Diagnostik und Therapie vorgenommen, um Entwicklungen über den gesamten Untersuchungszeitraum nachverfolgen zu können. Insgesamt finden sich 25 Fachbeiträge zur Therapie und 59 zur Diagnostik (Abb. 2).

Die Abbildung verdeutlicht, dass bis 1956 ein deutlicher Schwerpunkt auf der therapeutischen Anwendung von Radioisotopen in der Inneren Medizin lag. In der Etablierungsphase konzentrierte man sich auf den therapeutischen Einsatz radioaktiver Isotope in der Hoffnung, bisher schwer behandelbare Krankheiten zu therapieren. In den 1950er Jahren wurden daher unter anderem neue Ansätze zur Behandlung maligner hämatologischer Erkrankungen erprobt. Ferner setzten Internist:innen Jodisotope zur Behandlung verschiedener Schilddrüsenerkrankungen ein. Ab Mitte der 1950er Jahre verlagerte sich der Fokus: Therapeutische Interventionen traten zunehmend in den Hintergrund, während der Einsatz von Radioisotopen in der Diagnostik an Bedeutung gewann. Diese Entwicklung wurde maßgeblich durch den Import von Szintigrafen aus den USA vorangetrieben, die eine präzisere und effizientere bildgebende Diagnostik mit Radioisotopen in den Kliniken ermöglichten. Grundlagenforschung mit Radioisotopen an Patient:innen in Humanexperimenten und in Laboren waren über den gesamten Untersuchungszeitraum Teil der Forschung in internistischen Kliniken.

### Anwendung von Radioisotopen an Organsystemen

Für welche Organsysteme wurden Radioisotope zum Gegenstand klinisch-wissenschaftlicher Forschung in der Inneren Medizin? Hier zeigt die Auswertung deutliche Schwerpunkte: Mediziner:innen verwendeten Radioisotope vorwiegend in den Bereichen Blut und blutbildende Organe (61 Fachbeiträge) sowie Innere Sekretion/Hormone (58). Zudem fanden sich Veröffentlichungen zum Stoffwechsel (8), zur Leber (8) und dem Herz‑/Kreislaufsystem (7). Die Fokussierung auf das blutbildende System ist vor allem auf die frühe Verfügbarkeit von Radiophosphor zurückzuführen: Als erstes in der medizinischen Forschung verwendete Radioisotop wurde P^32^ als therapeutisches Mittel bei verschiedenen hämatologischen Erkrankungen erprobt. Für den Bereich Innere Sekretion/Hormone stand Radiojod als organspezifisches Isotop in Diagnostik und Therapie der Schilddrüse zur Verfügung. Die Übertragung diagnostischer Verfahren mit Radioisotopen auf andere Organsysteme fand im Zusammenhang mit der Implementierung von Szintigrafen an Universitätskliniken der Bundesrepublik statt.

### Orte internistischer Isotopenforschung

Die Auswertung lässt eindeutig Schwerpunkte an sechs Universitätskliniken in der Bundesrepublik erkennen. Über den gesamten Untersuchungszeitraum finden sich Veröffentlichungen von insgesamt 27 Orten, jedoch stammen 91 von 156 Fachbeiträgen und damit 58 Prozent aus sechs Medizinischen Universitätskliniken: Freiburg (22 Fachbeiträge), Berlin (16), Düsseldorf (16), Köln (15), Hamburg (12) und Göttingen (10). Die führende Rolle der Freiburger Klinik war auf das Engagement ihres Leiters Ludwig Heilmeyer (1899–1969) zurückzuführen. Bereits vor dem Zweiten Weltkrieg wurde er durch Georg von Hevesy auf die wissenschaftlichen Potenziale von Radioisotopen aufmerksam. Sein Mitarbeiter Friedhelm Odenthal trug in der Nachkriegszeit zum Aufbau eines eigenen Isotopenlabors an der Universitätsklinik Freiburg bei. Diagnostische und therapeutische Verfahren mit Radioeisen und Radiochrom bildeten dabei die Forschungsschwerpunkte (Feld & De Roo 2000: 36). Forschungsschwerpunkte an der Freien Universität Berlin stellten messtechnische Verfahren dar. Dies geschah in enger Zusammenarbeit zwischen Innerer Medizin, Strahleninstitut und der Auergesellschaft als industrieller Akteurin. Therapieversuche unternahm Irene Boll (1922–2013), eine der wenigen forschenden Internistinnen der Nachkriegszeit. Die Ergebnisse ihrer Behandlungsversuche mit Radioisotopen bei verschiedenen Leukämieformen publizierte sie nicht nur in medizinischen Fachzeitschriften, sondern präsentierte diese zudem auf dem Europäischen Kongress für Hämatologie (Boll 1956). Die Medizinische Akademie Düsseldorf verfügte seit 1957 über ein Betatron, das in der neu gegründeten Klinik für Medizinische Strahlenheilkunde eingesetzt wurde (Hitzer 2020: 359 f.). Während die Strahlentherapie in Düsseldorf einen Schwerpunkt darstellte, lag das Hauptinteresse der Inneren Medizin auf diagnostischen Verfahren zur Untersuchung der Schilddrüse. Unter der Leitung von Karl Oberdisse (1903–2002) konzentrierten sich seine Mitarbeiter:innen auf die Weiterentwicklung verschiedener diagnostischer Methoden mit Radiojod und markierten Isotopenverbindungen in vivo und in vitro. Ihre klinischen Erfahrungen präsentierten sie auf dem 69. Kongress der Deutschen Gesellschaft für Innere Medizin (Horster & Klein 1963). Das Isotopenlabor in Köln wurde auf Initiative des Internisten Hugo Wilhelm Knipping (1895–1984) eingerichtet. Die Forschungsinteressen des Direktors der Medizinischen Klinik Köln richteten sich vornehmlich auf die Diagnose und Behandlung von Krebserkrankungen, im speziellen Lungenkrebs. Knipping entwickelte beispielsweise in Zusammenarbeit mit dem Physikalischen Institut der Technischen Hochschule Aachen „ein mit 20 Szintillationszählern ausgerüstetes Gerät zur klinischen Anwendung“ (Knipping & Liese 1959: 71). Die Früherkennung von Lungenkrebs und die Darstellung der Herzdurchblutung stellten hier die Ziele der bildgebenden Diagnostik mit Radioisotopen dar. An der Universitätsklinik Hamburg kooperierten die Medizinische Klinik unter Leitung von Arthur Jores (1901–1982) und das Allgemeine Röntgeninstitut, wodurch verschiedene Therapiestudien bereits Anfang der 1950er Jahre bei Krankheiten des blutbildenden Systems und der Schilddrüse durchgeführt werden konnten. Neben dem Fokus auf die Therapie befassten sich vor allem Forscher:innen des Allgemeinen Röntgeninstituts mit der Weiterentwicklung der Diagnostik. So stammt der Radiojod-Zweiphasentest als diagnostische Innovation aus Hamburg (Feld & De Roo 2000: 38). In Göttingen befand sich nicht nur die zentrale Stelle für den Import radioaktiver Isotope, sondern die interdisziplinäre Zusammenarbeit zwischen der Inneren Medizin und der Medizinischen Physik führte auch zur Implementierung von Szintigrafen in der klinischen Diagnostik. Unter der Leitung von Rudolf Schoen (1892–1979) führte Paul Doering außerdem Humanexperimente durch, die erstens Einblicke in die Pathogenese verschiedener Leukämieformen geben sollten (Doering et al. 1957). Zweitens setzte er ein neu verfügbares Calciumisotop ein, um Normalwerte für die klinische Diagnostik zu gewinnen (Doering 1963).

Um hier ein kurzes Zwischenfazit zu ziehen, soll unterstrichen werden, dass im Untersuchungszeitraum eine kontinuierliche und in ihrer Zahl relativ konstante Forschung an und mit Radioisotopen in der internistischen Klinik zu erkennen ist. Internist:innen setzten ab den 1950er Jahren Isotope sowohl in der medizinischen Grundlagenforschung als auch in der klinischen Forschung ein, wobei zu Beginn die Therapie mit Isotopen den Schwerpunkt bildete. In den darauffolgenden Jahren kam es zu einer Ausweitung internistischer Forschungsvorhaben in Diagnostik und Grundlagenforschung, was auf die Verfügbarkeit von Szintillationszählern und Szintigrafen zurückzuführen ist. Eine deutliche Konzentration der Isotopenforschung zeigte sich dabei an sechs Universitätskliniken der Bundesrepublik: Freiburg, Berlin, Düsseldorf, Köln, Hamburg und Göttingen. Diese zeichneten sich durch eine hochwertige technische Ausstattung aus. Darüber hinaus profitierte die Forschung an diesen Standorten maßgeblich von Fördermitteln der DFG und des Bundesministeriums für Atomfragen. Ein weiterer Faktor war die interdisziplinäre Zusammenarbeit zwischen Innerer Medizin, Radiologie und Medizinischer Physik. Diese Schwerpunktbildung verdeutlicht, dass die Isotopenforschung nicht nur von technologischen und finanziellen Voraussetzungen abhing, sondern auch maßgeblich von interdisziplinären Kooperationen beeinflusst wurde.

Anschließend an diese quantitative Untersuchung zur Verbreitung von Radioisotopen führe ich eine qualitative Analyse spezifischer Forschungsprojekte durch: 1.) Zunächst werden Fachbeiträge analysiert, die über den therapeutischen Einsatz von Radioisotopen in der frühen Phase berichteten. 2.) Die Relevanz technologischer Innovationen sowie eine zunehmende Spezialisierung untersuche ich exemplarisch an Veröffentlichungen Medizinischer Universitätskliniken zum Einsatz von Szintigrafen in der Diagnostik und der Indikatormethode in der Laborforschung.

## Erprobung von Radioisotopen in der internistischen Klinik

Nachfolgend werden ausgewählte Beispiele des frühen Einsatzes von Radioisotopen in internistischen Universitätskliniken der Bundesrepublik vorgestellt. Fünf Fachbeiträge werden dazu näher betrachtet, die in den Anfangsjahren dieser Forschungen veröffentlicht und in denen radioaktive Isotope an Patient:innen eingesetzt wurden. Anhand dieser Beiträge lässt sich veranschaulichen, wie Internist:innen in Zusammenarbeit mit Radiolog:innen und Strahleninstituten radioaktive Isotope in Diagnostik und Therapie einsetzten. Gruppiert ist die Darstellung der Fachbeiträge nach den bereits erwähnten Schwerpunktbereichen Blut und blutbildende Organe sowie Innere Sekretion/Hormone.

### Radioisotope in der Therapie von Blutkrankheiten

Als erste hämatologische Erkrankung, die mit dem Radioisotop P^32^ erfolgreich behandelt werden konnte, erhielt Polycythaemia vera^5^ auch im deutschsprachigen Raum große Aufmerksamkeit. Bisherige Behandlungsverfahren wie Aderlass, frühe Chemotherapeutika und langanhaltende Röntgenbestrahlungen zeitigten nur mäßige Behandlungserfolge. Die ersten Veröffentlichungen von Therapieversuchen der Polycythaemia vera mit Radiophosphor an kleinen Patient:innengruppen wurden von den Universitätskliniken Freiburg und Hamburg vorgelegt.

Bereits 1949 – kurz nachdem Radioisotope in der Bundesrepublik verfügbar waren – begann Friedhelm Odenthal an der Medizinischen Klinik Freiburg Behandlungsversuche an 19 Patient:innen mit Radiophosphor. Der Präsentation der Ergebnisse zur therapeutischen Wirksamkeit gingen für die Klinik wichtige Fragen zur Organspezifität und Dosimetrie voran: Odenthal verdeutlichte, dass im klinischen Einsatz von Radiophosphor verschiedene Parameter für die Dosierung von Bedeutung waren, denn „die ‚biologische Halbwertszeit‘ ist geringer als die ‚physikalische Halbwertszeit‘“ (Odenthal 1951: 765). Dies liege vor allem daran, dass „[e]in Teil der verabfolgten Menge (in einzelnen Fällen bis zu 30 %) […] im Harn ausgeschieden“ (ebd.: 756) werde. Damit waren direkte Implikationen für die Behandlung verbunden, da die tatsächlich im Körper verbleibende Strahlenmenge schneller abnahm. Die Festlegung der therapeutischen Dosierung von Radiophosphor bei Patient:innen stellte zu Beginn eine Herausforderung dar, da es keine standardisierte Berechnungsgrundlage für die Anwendung von Radioisotopen gab (ebd.: 766). Dies führte zu einem weitgehend experimentellen Einsatz des Radiophosphors in der Klinik. Es bleibt jedoch unklar, ob die Patient:innen ausreichend über die experimentelle Natur der Therapie und die Notwendigkeit wiederholter Gaben radioaktiver Substanzen informiert waren.^6^ Erste Ergebnisse der Therapieversuche mit einer Nachbeobachtungszeit von zwei Jahren stellte Odenthal hinsichtlich der Verbesserung des Allgemeinbefindens der Erkrankten fest. Anhand von tabellarisch dargestellten Laborwerten zeigte er die Normalisierung des Blutbildes nach der therapeutischen Intervention mit P^32^ bei Polycythaemia vera (ebd.: 766 f.).

An der Medizinischen Universitätsklinik in Hamburg begannen Therapieversuche mit P^32^ in Zusammenarbeit mit dem Röntgeninstitut im Jahr 1950. An 17 Patient:innen wurde die Wirksamkeit und Sicherheit der Behandlung von Polycythaemia vera mit Radiophosphor erprobt. Die Normalisierung des Blutbildes nach wiederholter Gabe von P^32^ stellten die Forscher^7^ tabellarisch dar: „Alle Patienten, die zum Teil seit Jahren wiederholt bzw. fortgesetzt einer Röntgen- oder Phenylhydrazintherapie unterzogen wurden, sind praktisch beschwerdefrei und brauchten bis jetzt nicht wieder behandelt werden.“ (Goldeck et al. 1952: 29) Hinsichtlich der therapeutischen Dosierung machten sie konkrete Angaben, die sich nach „Alter, Allgemeinzustand, Blutbild und Vorbehandlung“ richteten, wobei eine Initialdosis und „nach zwei bis drei Monaten je nach Erfolg eine weitere, eventuell höhere Dosis“ (ebd.: 28) gegeben wurde.

Während Odenthal seinen Fokus auf die Ermittlung einer therapeutisch wirksamen Dosis richtete, untersuchten die Mediziner aus der Medizinischen Klinik und dem Strahleninstitut Hamburg die Wirkung von Radiophosphor auf das blutbildende System. Klinische Fragestellungen zur Organspezifität übertrugen sie ins Labor. In Tierexperimenten an drei Gruppen von Ratten erforschten sie die spezifische Wirkungsweise auf die Bildung neuer Blutkörperchen. „Wir versuchten tierexperimentell die erythropoetische Hemmung durch die β‑Strahlung des Radiophosphors an den Reticulocytenverschiebungen der Laborratte sichtbar zu machen.“ (Ebd.: 29) Der therapeutische Einsatz von Radiophosphor stieß weitere Laborforschung an, die zu einem vertieften Verständnis von Strahlenwirkung auf das blutbildende System führte. Aus den Tierexperimenten leiteten die Hamburger Forscher zudem für „die praktische Therapie“ ab, „daß der regeneratorische Reiz eines Aderlasses am besten durch eine synchrone Radiophosphorgabe abgefangen wird“ (ebd.: 30). In diesem Fachbeitrag wird das dynamische Wechselspiel zwischen grundlagenorientierter und klinischer Forschung in der Inneren Medizin deutlich. Der therapeutische Einsatz von Radiophosphor führte zu Forschungsfragen, die ins Labor übertragen wurden. Dabei lieferten die Experimente nicht nur Erkenntnisse zur Strahlenwirkung, sondern warfen auch neue Fragen zum therapeutischen Vorgehen mit radioaktiven Isotopen auf.

### Radiojod in der Diagnostik und Therapie im Bereich der Inneren Sekretion

Die Untersuchung und Behandlung von Schilddrüsenerkrankungen, vornehmlich Hyperthyreosen, mit Radiojod war der zweite Bereich, in dem radioaktive Isotope in der internistischen Klinik Anfang der 1950er Jahre erprobt wurden. Die folgenden drei Beispiele wurden ausgewählt, um zum einen die Erweiterung diagnostischer Techniken mit der Tracermethode und zum anderen die Etablierung der Radiojodtherapie in der Klinik zu diskutieren.

Als notwendige Bedingung für den Einsatz von Radioisotopen in der Inneren Medizin sind Geiger-Müller-Zählrohre^8^ als technische Instrumente in der Klinik gesondert zu betrachten. Ein Fachbeitrag der Freien Universität Berlin untersuchte diese Messtechnik an Patient:innen mit Schilddrüsenerkrankungen. Im Zentrum der Analyse standen die technischen Rahmenbedingungen und die exakte Anwendung von Radiojodmessungen in der Diagnostik, wie sie zu Beginn der 1950er Jahre praktiziert wurden. Der Einsatz von Geiger-Müller-Zählrohren erforderte eine möglichst standardisierte Messumgebung, da bereits geringe Abweichungen bei der Einstellung und Zentrierung die Messergebnisse beeinflussen konnten (Billion et al. 1951: 306). Neben umfassenden technischen Kenntnissen war die interdisziplinäre Zusammenarbeit unterschiedlicher Fachbereiche eine zentrale Voraussetzung für die Implementierung neuer diagnostischer Verfahren mit radioaktiven Isotopen in der Klinik. So kooperierten der Strahlentherapeut Hans Billion und der Internist Klaus Oeff bei der Einführung des Radiojodtests.

Im Anschluss an die Darstellung technischer Bedingungen der Diagnostik mit Radioisotopen fand ein Vergleich mit anderen Methoden statt: Ziel war es zunächst, den Wert des Radiojodtests für die Diagnose von Schilddrüsenerkrankungen im Vergleich zu den bisherigen diagnostischen Techniken zu bestimmen. Gesunde Versuchspersonen erhielten eine Tracerdosis radioaktiven Jods, um Normalwerte der Jodspeicherung zu ermitteln. Dieses Humanexperiment an gesunden Menschen war als Form der Grundlagenforschung in der Klinik entscheidend für die Implementierung neuer diagnostischer Techniken, um Vergleichswerte für die Diagnostik zu erhalten. Im Anschluss führten die Forscher den Radiojodtest an 65 Patient:innen durch, bei denen eine Störung der Schilddrüsenfunktion vermutet wurde, um den diagnostischen Wert für die Klinik zu ermitteln. Zwei Formen des Radiojodtests lieferten dabei Aufschluss über die Schilddrüsenfunktion – erstens die direkte Messung an Erkrankten nach Verabfolgung von J^131^. Dazu nahmen diese an acht Messungen innerhalb von 48 Stunden teil und lagen je Messvorgang zehn Minuten unter der Messvorrichtung. Die hohe Anzahl an Messvorgängen innerhalb von zwei Tagen ging mit Belastungen für die Kranken einher, was jedoch vonseiten der Forscher nicht reflektiert wurde. Zweitens ermittelten die Mediziner die Menge des im Urin ausgeschiedenen Radiojods. Auf Grundlage dieser Messergebnisse ließ sich „eine exakte Aussage über den Funktionszustand der Schilddrüse“ treffen. So resümierte das Berliner Forschungsteam hinsichtlich der diagnostischen Validität der neuen Messtechnik: „Gerade bei der Klärung unklarer Krankheitsbilder erwies sich der RJ-Test als überlegen gegenüber anderen diagnostischen Verfahren, speziell gegenüber der Grundumsatzbestimmung.“ (Ebd.: 307) Dieses neue Verfahren erwies sich als wertvolle Erweiterung für die Differenzialdiagnostik in der Klinik: Schilddrüsenüberfunktionen konnten anhand der Jodspeicherungskurve von anderen Erkrankungen, die eine ähnliche Symptomatik aufwiesen, unterschieden werden.

Zudem ermöglichte der Radiojodtest das Studium von Stoffwechselvorgängen in der Klinik (ebd.: 309). Dies ließ sich nicht nur für die Diagnostik im Kontext von Störungen des Muskel- und Fettstoffwechsels in Abgrenzung zur Hyperthyreose verwenden, sondern ebenso als Verfahren zur Erforschung pathophysiologischer Zusammenhänge zwischen endokrinem System und Stoffwechselprozessen. Das diagnostische Verfahren mit Radiojod war somit auf zweifache Weise für die internistische Klinik wichtig: In der Diagnostik lieferte es valide Ergebnisse für das Vorliegen einer Hyperthyreose. Im Rahmen pathophysiologischer Forschung in der Klinik an Patient:innen bestand die Relevanz in der Erweiterung des Verständnisses endokriner Störungen und deren Unterscheidung von Stoffwechselstörungen.

Im Rahmen zweier Veröffentlichungen der Medizinischen Universitätsklinik Hamburg in Zusammenarbeit mit dem Allgemeinen Röntgeninstitut wurden therapeutische Ansätze zur Verwendung von Radiojod bei Schilddrüsenüberfunktion vorgestellt. Einerseits zielte die Forschung auf die Ermittlung einer Standarddosis für die Behandlung mit radioaktiven Isotopen. Andererseits debattierten Forscher:innen in den 1950er Jahren über die Einheit, in der die therapeutische Dosierung eines Radioisotops gegeben werden sollte. Für den klinischen Einsatz bedeutete dies, dass eine andere Berechnung der Strahlendosis, die „nach der Ionisationswirkung im Gewebe bestimmt“ (Horst 1951: 1053) und für Patient:innen individuell anhand der Schilddrüsengröße zu ermitteln war.

Die erste der beiden Forschungsarbeiten thematisierte zunächst Fragen der Dosimetrie. Im Anschluss stellte Wolfgang Horst die Behandlung von 25 Patient:innen der Medizinischen Klinik Hamburg mit Radiojod vor. Als klinische Fragen erörterte er die Problematik der Indikationsstellung gegenüber bereits etablierten Verfahren wie chirurgischer Entfernung der Schilddrüse, pharmakologischer Therapie mit Thyreostatika oder Röntgenbestrahlung (ebd.: 1053). In der Auseinandersetzung mit bisherigen Forschungsergebnissen zur Radiojodtherapie aus den USA und den Risiken der bestehenden Verfahren begründete er die Auswahl: Die Teilnehmer:innen des therapeutischen Versuchs waren entweder bereits erfolglos mit Thyreostatika behandelt worden, erlitten ein Rezidiv nach Röntgentherapie oder der Allgemeinzustand war zu schlecht, um die Risiken eines chirurgischen Eingriffs eingehen zu können (ebd.: 1055). Die anschließende Behandlung mit einer individuell ermittelten therapeutischen Dosis Radiojod „führte bei 18 der 25 Patienten schon nach der ersten Radiojoddosis zum Erfolg, der bei 5 weiteren Patienten erst nach der 2. bzw. 3. Radiojoddosis befriedigte“ (ebd.: 1056). Die Radiojodtherapie erwies sich als Erweiterung der klinischen Behandlungsmöglichkeiten – insbesondere in Fällen, in denen die bisherigen Therapieoptionen keine dauerhafte Verbesserung erzielten.

Bereits im Jahr 1954 lag eine Übersichtsarbeit von Wolfgang Horst und Friedrich Kuhlencordt zur Wirksamkeit der Behandlung von Hyperthyreose mit J^131^ an insgesamt 150 Patient:innen vor. Die Ergebnisse zeigten, dass die Radiojodtherapie bei 88 Prozent der Fälle (132 Patient:innen) als erfolgreich beurteilt wurde, wobei es auch zu einer Verkleinerung der Struma kam (Horst & Kuhlencordt 1954: 441). Neben der therapeutischen Wirksamkeit untersuchten Horst und Kuhlencordt auch den Jodstoffwechsel und resümierten, dass „[d]ie Ergebnisse der Radiojodstoffwechseluntersuchung nach erfolgreicher Jod-^131^-Therapie […] für das Bestehen von zwei pathogenetisch verschiedenartigen Gruppen der Schilddrüsenüberfunktion [sprechen]“ (ebd.: 485). Anhand der Nachuntersuchungen zeigte sich, dass unterschiedliche Mechanismen der Krankheit vorlagen.

Die hier genauer vorgestellten Beispiele zeigen, dass Forschungsvorhaben zu Beginn der 1950er Jahre auf die Erweiterung des diagnostischen und therapeutischen Repertoires in der Klinik ausgerichtet waren, wobei eine Übertragung relevanter Forschungsfragen zur Strahlenwirkung im Organismus aus der Klinik in die Grundlagenforschung stattfand. Dabei liefen Humanexperimente zur Ermittlung von Vergleichswerten, Forschungsfragen der Dosimetrie und pathophysiologische Untersuchungen an Kranken parallel zum klinischen Einsatz von Radioisotopen in Diagnostik und Therapie. Laborforschungen lieferten zudem neue Erkenntnisse zur Wirkungsweise von Radioisotopen auf Organsysteme, die für therapeutische Anwendungen in der Klinik genutzt wurden. Die Wissensproduktion wurde maßgeblich durch die interdisziplinäre Zusammenarbeit zwischen Innerer Medizin und Radiologie geprägt. Die Implementierung von Radioisotopen und technologischen Innovationen in der Klinik erforderte sowohl medizinisches als auch physikalisches Wissen. Dieses wurde in Lehrbüchern aufbereitet und einem breiten Publikum zugänglich gemacht. Exemplarisch hierfür steht das 1953 von dem Internisten Herbert Schwiegk (1906–1988) herausgegebene Werk *Künstliche radioaktive Isotope in Physiologie, Diagnostik und Therapie*. Dieses Werk zeigt eindrucksvoll, wie das Wissen aus verschiedenen Disziplinen zusammengeführt wurde. Es bündelt den damaligen Kenntnisstand zur Radioisotopenforschung der einzelnen Fachbereiche. So finden sich Ausarbeitungen zur Therapie in der Hämatologie (Heilmeyer & Odenthal 1953) und Diagnostik in der Endokrinologie (Schwiegk & Lang 1953) aus der Inneren Medizin neben Beiträgen zum Strahlenschutz und der Einrichtung von Isotopenlaboratorien aus der Biophysik (Hug & Muth 1953).

## Technikgetriebene Wandlungsprozesse in Medizinischen Universitätskliniken

Ab Mitte der 1950er Jahre differenzierten sich Forschungsvorhaben mit radioaktiven Isotopen in der Inneren Medizin zunehmend. Dies war zum einen auf die verstärkte Verfügbarkeit technischer Instrumente wie Szintillationszähler und Szintigrafen zurückzuführen. Zum anderen begünstigten interdisziplinäre Kooperationen zwischen medizinischen Universitätskliniken und spezialisierten Isotopenlaboratorien die Bearbeitung komplexer Forschungsfragen an der Schnittstelle zwischen Klinik und Labor.

### Diagnostik mit Szintigrafen in der Inneren Medizin

Unverzichtbare Voraussetzung der Anwendung von Isotopen in der internistischen Klinik waren ausgefeilte Instrumente zur Registrierung der Strahlenwirkung. Szintillationszähler ermöglichten im Gegensatz zu Geiger-Müller-Zählrohren die genaue Messung γ‑strahlender Radioisotope. Verfügbar wurden Szintillationszähler vorwiegend über den US-amerikanischen Markt; insbesondere die Firma Tracerlab aus Boston belieferte Forscher:innen mit diesem Instrument. Die Anschaffungskosten dieser Geräte wurden teilweise aus den Mitteln der DFG finanziert, in der Inneren Medizin vornehmlich aus den bewilligten Sachmitteln im Normalverfahren Medizin.^9^ Ferner stellte das 1955 gegründete Bundesministerium für Atomfragen Fördermittel für die Einrichtung zentraler Isotopenlaboratorien an Universitäten zur Verfügung (von Schwerin 2015: 361).

Szintigrafen, die ab Mitte der 1950er Jahre in internistischen Kliniken der Bundesrepublik verfügbar waren, bestanden aus mehreren Teilen: 1.) Aus einem Kristall (Szintillator), um den Effekt der Szintillation – die Auslösung eines Lichtblitzes – durch das Auftreffen radioaktiver Strahlung auf den Kristall zu nutzen. 2.) Aus einem Photomultiplier – das heißt einer Elektronenröhre, die Photonen in elektrische Signale umwandelt. 3.) Aus einem Verstärker, der die Energieimpulse in der Messanordnung weiterleitet sowie 4.) Bleiabschirmungen und Bleiblende (Doering 1956a: 386 f.). Die Ergänzung der Szintillationszähler um eine automatische Schreibvorrichtung, die aus Elektrode und Metallfolie bestand, um die Impulse als Szintigramme aufzeichnen zu können, ermöglichte den breiten Einsatz in der bildgebenden Diagnostik.

Dieses komplexe Instrument stellte eine wichtige technische Erweiterung für die klinische Diagnostik dar, wie nachfolgend anhand exemplarisch ausgewählter Fachbeiträge analysiert wird. So wurde an der Medizinischen Universitätsklinik Göttingen 1956 in Zusammenarbeit mit dem Institut für Medizinische Physik ein Szintigraf für die klinische Diagnostik installiert. Paul Doering berichtete in der Zeitschrift *Deutsches Archiv für klinische Medizin* in zwei separaten Beiträgen über die technischen Voraussetzungen und erste diagnostische Anwendungen an Patient:innen. Dieses Verfahren war für die Innere Medizin ein Fortschritt für die bildgebende Diagnostik mit Radioisotopen: „Während die Bestimmung biologischer Größen an Menschen mit reinen β‑Strahlern (z. B. C^14^, P^32^, S^35^) *in vitro*-Messungen voraussetzt, gestattet die Untersuchung mit γ‑Strahlen emittierenden Radioisotopen, das Schicksal solcher Elemente auch *in vivo* zu verfolgen.“ (Doering 1956a: 384) Die Messtechnik wurde in der internistischen Klinik erstens dazu eingesetzt, Darstellungen der normalen und pathologisch veränderten Schilddrüse zu gewinnen. Zweitens konnte durch die Szintigrafie eine Differenzierung der verschiedenen pathologischen Veränderungen anhand der vorliegenden Szintigramme vorgenommen werden. Dies war für die Unterscheidung von Hyperthyreosen, verschiedenen Formen der Struma und Schilddrüsenkarzinomen in der Klinik von großer Bedeutung. Drittens eignete sich der Szintigraf dazu, eine exakte Radiojoddosis im Rahmen der Therapie von Schilddrüsenerkrankungen zu ermitteln (Doering 1956b: 410 ff.). Die in-vivo-Verfolgung des Therapieeffekts durch die Szintigrafie ermöglichte in der internistischen Klinik eine genaue Dosierung der Radiojodgaben und die Ausarbeitung eines Therapieplans, der sowohl Über- als auch Unterdosierung von Radioisotopen verhinderte. Die exakte Dosierung von kernchemischen Pharmaka war bis Mitte der 1950er Jahre eines der vielbehandelten Forschungsprobleme. Die Verfügbarkeit von Szintillationszählern und der Aufbau von Szintigrafen waren Meilensteine für die Radioisotopenforschung und den Einsatz an Patient:innen in der internistischen Klinik. Diese Messtechnik wurde in der Folgezeit auf verschiedene Organsysteme übertragen – darunter Leber (Doering 1957), Niere (zum Winkel et al. 1961) und Milz (Fischer & Wolf 1963).

### Laborforschung in internistischen Kliniken mit der Indikatormethode

Für die Erforschung physiologischer und pathophysiologischer Fragestellungen in der Inneren Medizin stellte die Tracermethode, im deutschsprachigen Raum auch Indikatormethode genannt, eine erhebliche Erweiterung der Methodik dar. Der Einsatz radioaktiver Isotope als Indikatoren ermöglichte tiefere Einblicke in (patho-)physiologische Prozesse: Durch die Verabreichung minimaler Isotopenmengen konnten Stoffwechselvorgänge, Neubildungsraten von Zellen und die Abbaugeschwindigkeit spezifischer Moleküle auf atomarer Ebene untersucht werden (vgl. Hevesy 1957). Welche konkreten Forschungsprojekte in internistischen Kliniken mit der Indikatormethode durchgeführt wurden und welche Formen der Wissensproduktion damit einhergingen, soll anhand von zwei Beispielen aus Freiburg und Mainz veranschaulicht werden. Zugleich soll anhand dieser exemplarischen Fachbeiträge verdeutlicht werden, wie Transferprozesse zwischen Labor und Klinik abliefen.

Ein ausgewähltes Beispiel zur Übertragung der Indikatormethode kam 1960 aus der Medizinischen Universitätsklinik Freiburg. In einem Tierexperiment wurde die Hypothese untersucht, ob pathophysiologische Zusammenhänge zwischen der Schilddrüse und dem erythrozytärem System bestehen. Die Freiburger Forscher:innen ermittelten zunächst die Lebenszeit von Erythrozyten bei gesunden Versuchstieren mittels der Radiochrommethode (Keiderling & Frank 1960: 382). Im Experiment wurde Blut entnommen, labortechnisch bearbeitet und mit radioaktivem Chrom markiert, bevor es den Versuchstieren injiziert wurde. Über einen Zeitraum von sieben Tagen erfolgten in festgelegten Abständen Blutentnahmen, deren radioaktive Strahlung anschließend mit einem Szintillationszähler gemessen wurde. Die so gewonnenen Messdaten ermöglichten die Berechnung des Anteils an Erythrozyten zu verschiedenen Zeitpunkten. Die Berechnungen erfolgten in Zusammenarbeit mit dem Physikalischen Institut der Universität Freiburg. Im nächsten Schritt wurde die Hypothese geprüft, ob pathologische Veränderungen der Schilddrüse den Erythrozytenstoffwechsel beeinflussen. Dazu wurden Schilddrüsenerkrankungen wie Hypothyreose durch Radiojod und Hyperthyreose durch Thyroxin induziert. Mithilfe der Cr^51^-Methode wurden Veränderungen der Erythrozytenlebenszeit bei den Versuchstieren festgestellt. Diese Untersuchung diente dem Nachweis pathophysiologischer Zusammenhänge zwischen Schilddrüse und Erythrozytenstoffwechsel (ebd.: 380 f.).

In einem weiteren Schritt wurde die Nützlichkeit der Radiochrommethode für die hämatologische Forschung im Labor überprüft: Die Markierung von Erythrozyten mit Radiochrom ermöglichte eine detaillierte Erfassung hämolytischer Prozesse und erwies sich damit als Weiterentwicklung zum Studium des blutbildenden Systems. Die Anwendbarkeit der Cr^51^-Methode blieb jedoch nicht aufs Labor beschränkt, sondern erschien in der Diagnostik geeignet „zum quantitativen Nachweis eines gesteigerten Blutabbaues bei den verschiedensten hämatologischen Störungen“ (ebd.: 384). Mit der Implementierung der Radiochrommethode in der klinischen Anwendung ging eine Erweiterung der diagnostischen Möglichkeiten für Erkrankungen des blutbildenden Systems einher.

Wie verlief die interdisziplinäre Zusammenarbeit zwischen der Inneren Medizin und spezialisierten Isotopenabteilungen? Das nachfolgend vorgestellte Projekt der Universitätsklinik Mainz veranschaulicht exemplarisch die Übertragung klinischer Forschungsfragen ins Labor: In einem Kooperationsprojekt zwischen der Mainzer Klinik und der Abteilung für Strahlenbiologie und Isotopenforschung am Strahleninstitut Marburg wurden iatrogene Schädigungen im Labor untersucht. Die erste Veröffentlichung befasste sich mit der Entwicklung eines in-vitro-Systems zur Erforschung der Zusammenhänge zwischen Nebenwirkungen pharmazeutischer Präparate und dem blutbildenden System. Ziel war es, mittels radioaktiv markiertem Leucin (C^14^-Leucin) den Einfluss verschiedener Substanzen auf den Einbau von Aminosäuren zu untersuchen (Mainzer et al. 1965). Die zweite Publikation behandelte den Einsatz des in-vitro-Systems, um die Auswirkungen verschiedener Pharmaka – vorrangig Antibiotika und Corticosteroide – hinsichtlich des Einbaus proteingebundener Aminosäuren in Retikulozyten zu untersuchen.

Für beide Wirkstoffgruppen stellten die Forscher^10^ eine Hemmung des Einbaus von C^14^-Leukin fest (Mainzer & Schaumlöffel 1965: 330). Die pharmakologischen Präparate hatten also negative Auswirkungen auf das blutbildende System. Die Erforschung der Nebenwirkungen pharmazeutischer Präparate veranschaulicht exemplarisch, wie spezielle Forschungsfragen aus der klinischen Tätigkeit entstehen: Die Beobachtung unerwünschter Wirkungen neuer Arzneimittel auf Patient:innen warf Fragestellungen für die laborexperimentelle Forschung auf.

Die Beispiele belegen, dass sich in den 1960er Jahren eine zunehmende Komplexität der Verwendung von Radioisotopen in der Inneren Medizin abzeichnete: Forscher:innen setzten Radioisotope nicht nur als Indikatoren in Laborexperimenten ein, sondern nutzten diese auch zur Veränderung von Tiermodellen in pathophysiologischen Experimenten. Markierungstechniken aus dem Labor fanden Eingang in die klinische Diagnostik. In interdisziplinären Forschungsprojekten zwischen Theoretischen Instituten und internistischen Kliniken nutzten Mediziner:innen die Indikatormethode zur Klärung relevanter klinischer Problemstellungen.

## Zusammenfassung und Ausblick

Die Forschungsfragen nach der Bedeutung technikgetriebener Wandlungsprozesse für die internistische Klinik können nun vor dem Hintergrund der dargestellten Ergebnisse wie folgt beantwortet werden: Ausgehend von einer systematischen Analyse dreier für die Innere Medizin relevanter Fachzeitschriften ist festzuhalten, dass Internist:innen Radioisotope in Diagnostik und Therapie einsetzten, sobald sie in der Bundesrepublik verfügbar waren. Dabei ließen sich Schwerpunkte der Forschung mit Radioisotopen in internistischen Einrichtungen in bestimmten Phasen identifizieren: In den frühen 1950er Jahren lag der Fokus auf der Therapie bisher schwer behandelbarer Krankheiten des blutbildenden Systems und der Schilddrüse. Während das erweiterte Handlungsspektrum in der Klinik begeistert aufgenommen wurde, fehlten kritische Perspektiven zur Anwendung von Radioisotopen an Patient:innen: Weder wurden forschungsethische Überlegungen zu Humanexperimenten mit Radioisotopen diskutiert, noch reflektierten die Forscher:innen die Belastungen, welche mit dem diagnostischen und therapeutischen Einsatz von radioaktiven Isotopen für die Erkrankten einhergingen. Erst Mitte der 1950er Jahre nahm der therapeutische Enthusiasmus ab, als die Nebenwirkungen von Radioisotopen zunehmend in den Fokus rückten (Doering 1956c, Emrich & Keiderling 1963).

Mit der Einführung technologischer Neuerungen – insbesondere Szintillationszählern und Szintigrafen – erweiterte sich ab Mitte der 1950er Jahre das diagnostische Spektrum in der internistischen Klinik. Die Einführung von Szintigrafen führte zu einer deutlichen Verschiebung der Forschungsschwerpunkte hin zur Diagnostik mittels Tracermethode. Mehrere Faktoren trieben diese Entwicklung voran: Einerseits konnten die anfangs hochgesteckten Erwartungen an die therapeutische Wirksamkeit radioaktiver Isotope nur bei einer begrenzten Anzahl von internistischen Erkrankungen erfüllt werden. Andererseits eröffneten technische Instrumente die Möglichkeit präziser bildgebender Diagnostik. Unterstützt durch gezielte Fördermaßnahmen der DFG und des Bundesministeriums für Atomfragen verlief der Einbau von Szintigrafen in Medizinischen Kliniken in enger Zusammenarbeit zwischen Internist:innen und der Medizinischen Physik. Erwähnenswert ist außerdem, dass sich die selektive Strahlenbehandlung mit Radioisotopen in speziell eingerichtete Strahlenkliniken verlagerte, womit eine Ausdifferenzierung und Spezialisierung der therapeutischen Anwendung von Radioisotopen einherging. Neben dem Fokus auf Diagnostik mit Radioisotopen ist für die 1960er Jahre eine zunehmende Ausdifferenzierung zu verzeichnen: Internist:innen übertrugen Markierungstechniken mit radioaktiven Isotopen aus der Laborforschung in die Klinik. Darüber hinaus intensivierte sich die interdisziplinäre Zusammenarbeit zwischen internistischen Einrichtungen, Strahleninstituten und neu eingerichteten Isotopenlaboratorien.

Die Isotopenforschung in Medizinischen Kliniken der Nachkriegszeit lässt nicht zuletzt ein dynamisches Wechselspiel von Grundlagenforschung und klinischer Forschung erkennen: Wie an mehreren Beispielen aufgezeigt, entwickelten sich grundlagenorientierte Forschungsfragen direkt aus dem Einsatz von Radioisotopen an Patient:innen. Internist:innen führten daraufhin Untersuchungen zur Dosimetrie und Organspezifität in der Klinik durch. Gleichzeitig lösten klinische Beobachtungen neue grundlagenorientierte Fragestellungen aus, die zu einem besseren Verständnis des Verbleibs und der Wirkungsweise radioaktiver Isotope in den Organsystemen führten. Therapieversuche warfen weitere Fragen auf, die wiederum das Wissen über die Wirkmechanismen radioaktiver Isotope vertieften. Diagnostische Anwendungen ermöglichten die Formulierung neuer Hypothesen zur Pathophysiologie und Pathogenese unmittelbar an Patient:innen. Tierexperimente, die auf die Klärung physiologischer und pathophysiologischer Fragen abzielten, erleichterten die Übertragung laborexperimenteller Methoden in die klinische Diagnostik.

Die Forschung mit Radioisotopen in der Inneren Medizin war insgesamt durch die enge Verbindung von Labor und Klinik charakterisiert – ein Ansatz, der in der heutigen Debatte um Translationale Medizin (TM) von zentraler Bedeutung ist.^11^ Auch wenn der Begriff „Translationale Medizin“ erst Ende der 1990er Jahre aufkam, weisen neuere Untersuchungen darauf hin, dass die Interaktion zwischen Labor und Klinik in deutlich längeren Entwicklungslinien gesehen werden kann (Worboys et al. 2021). In diesem Sinne kann die Forschung an und mit Radioisotopen in der Inneren Medizin als Translationale Medizin *avant la lettre* betrachtet werden: Im ständigen Austausch zwischen kliniknaher Grundlagenforschung und klinischer Forschung zirkulierten Forschungsfragen, Erkenntnisse und technologische Instrumente zwischen Labor und Klinik. So führte etwa der therapeutische Einsatz von Radioisotopen zu neuen experimentellen Fragen, die die Strahlenwirkung auf verschiedene Organsysteme beleuchteten und Impulse für die Therapie gaben. Auch die Anwendung der Tracermethode in der klinischen Diagnostik lieferte neue Einblicke in pathophysiologische Mechanismen und trug damit zur Erforschung von Krankheitsprozessen bei. Zunehmend wurden Fragestellungen aus der klinischen Praxis in spezialisierte Isotopenlabore übertragen, wodurch sich die interdisziplinäre Kooperation intensivierte. Durch diese enge Zusammenarbeit konnten Fragen aus der Klinik systematisch in experimentelle Untersuchungen überführt werden, um die Ergebnisse anschließend für diagnostische und therapeutische Verfahren in Medizinischen Kliniken einzusetzen.

## Danksagung

Mein Dank gilt den anonymen Gutachter:innen und der Redaktion von NTM für ihre wertvollen Anmerkungen und Hinweise. Für fruchtbare Diskussionen im Projektteam danke ich Hans-Georg Hofer, Heiner Raspe, Vina Zielonka und Annika Berger. Mein Dank gilt zudem Nina Lüke, die mich bei der empirischen Auswertung unterstützt hat.

## Footnotes

1 Der vorliegende Beitrag ist im Rahmen des von der DFG geförderten Projekts „Innere Medizin als Wissenschaft – Wissenschaftlichkeiten der klinischen Medizin, 1945–1965“ unter Leitung von Hans-Georg Hofer in Kooperation mit Heiner Raspe (HO 4726/5 und RA 314/15) als Fallstudie entstanden. Im Fokus des Projekts stehen die Vielfalt und Heterogenität der Methoden, theoretischen Ansätze und Forschungspraktiken unter dem Dach der internistischen Klinik in den ersten beiden Nachkriegsjahrzehnten. Die Entwicklung der Inneren Medizin als Wissenschaft soll anhand fünf zentraler Forschungsfelder („Wissenschaftlichkeiten“) systematisch untersucht werden. Diese umfassen kliniknahe Grundlagenforschung, evaluative klinische Forschung, anthropologisch-psychosomatische Forschung, sozialmedizinische Forschung und allgemeine klinische Praxeologie. Kontroversen um die Psychosomatik in der Inneren Medizin wurden bereits im Kontext epistemologischer Debatten der direkten Nachkriegszeit behandelt (Hofer & Roelcke 2021). Ebenso sind Wandlungsprozesse der evaluativen klinischen Forschung in der Bundesrepublik analysiert worden: Die Methodenlehre klinisch-therapeutischer Forschung des Bonner Internisten Paul Martini (1889–1964) hatte zum Ziel, eine wissenschaftlich fundierte Grundlage für therapeutisches Handeln zu schaffen (Raspe et al. 2023a; Raspe et al. 2023b; Zielonka et al. 2023; Hofer et al. 2024). Die Grundlagenforschung in der Inneren Medizin und ihre Verbindung zur klinischen Forschung ist dagegen bisher kaum erforscht. Auch eine systematische Analyse technikgetriebener Wandlungsprozesse für die internistische Klinik am Beispiel der Isotopenforschung wurde bislang noch nicht unternommen.

2 Unter einem Isotop versteht man Atome desselben Elements, die im Atomkern dieselbe Protonenzahl aufweisen, sich aber in der Neutronenzahl unterscheiden. Somit bleiben Elemente der Ordnungszahl nach gleich, haben aber eine andere Massenzahl. Bekannt sind vier Kategorien von Isotopen: Primordial, kosmogen, radiogen und künstlich. Während die ersten drei natürlich vorkommenden Isotope für Forschungen unter anderem in der Geologie und Archäologie von Relevanz sind, fand mit der Herstellung künstlicher radioaktiver Isotope in kernphysikalischen Großgeräten (Zyklotron, Kernreaktor) die Übertragung dieser kernchemischen Technik in die medizinische Forschung statt. Radioaktive Isotope emittieren im Zerfallsprozess verschiedene Arten von Strahlung (α-, β‑ und γ‑Strahlung).

3 Bei der Tracermethode injizieren Forscher:innen radioaktive Isotope, um die Anreicherung oder die Spur von chemischen Stoffen im menschlichen oder tierischen Organismus nachzuverfolgen. In der Medizin kamen zunächst organspezifische Radioisotope wie Radiojod (J^131^) zum Einsatz. Dabei nutzten Mediziner:innen die Eigenschaft der Schilddrüse, Jod anzureichern, um diagnostische Verfahren zu entwickeln: Patient:innen wurde eine geringe Dosis J^131^ verabreicht, das sich in der Schilddrüse anreicherte und mit messtechnischen Instrumenten registriert werden konnte.

4 Zudem dienen einschlägige Veröffentlichungen zur wichtigen technologischen Neuerung der Szintigrafie aus der Zeitschrift *Deutsches Archiv für klinische Medizin* als Quellengrundlage.

5 Patient:innen, die an Polycythaemia vera erkrankt sind, weisen eine pathologische Vermehrung roter Blutkörperchen (Erythrozyten) auf, die mit diversen Beschwerden einhergeht.

6 Ein solch intransparentes Vorgehen hinsichtlich der Aufklärung von Versuchspersonen in der diagnostischen und therapeutischen Forschung der Inneren Medizin in der Nachkriegszeit ist nicht auf diesen Fachbeitrag beschränkt, sondern charakteristisch für die Veröffentlichungen in medizinischen Fachzeitschriften. In keiner der Publikationen aus internistischen Kliniken ist ersichtlich, ob Proband:innen über die möglichen Gefahren unterrichtet noch ob sie umfassend über die jeweilige Intervention aufgeklärt wurden.

7 In der Veröffentlichung des Therapieversuchs waren nur männliche Forscher gelistet.

8 Die Messtechnik von radioaktiven Substanzen mit Zählrohren wurde in den 1920er Jahren von den Physikern Hans Geiger (1882–1945) und Walther Müller (1905–1979) erforscht. Das seit 1928 verfügbare Geiger-Müller-Zählrohr ist folgendermaßen aufgebaut: Ein Metallzylinder dient als Gehäuse, ein Eintrittsfenster und ein dünner Metalldraht bilden das Grundgerüst. Der Metallzylinder wird mit einem Edelgas befüllt, vakuumdicht verschlossen und unter Spannung gesetzt, wobei der Metalldraht im Inneren positiv geladen ist. Diese Messvorrichtung wurde an einen Zähler angeschlossen, um die Impulse zu registrieren, die entstehen, wenn radioaktive Strahlung eintritt. Diese verursacht die Freisetzung von Sekundärelektronen durch Ionisation aus dem Edelgas, die als Zählimpulse registriert werden können. Es waren verschiedene Typen von Geiger-Müller-Zählrohren für die Anwendung in der Medizin verfügbar (vgl. Schmeiser 1951: 719 f.).

9 Zudem ermöglichten Mittel aus dem Schwerpunktprogramm Radiologie die Ausstattung von theoretischen Instituten und klinischen Abteilungen mit aufwendiger Labortechnik (Schwerin 2015: 332).

10 Auch in diesem Forschungsprojekt waren nur männliche Forscher verzeichnet.

11 Siehe zum Konzept der Translationalen Forschung beziehungsweise Translationalen Medizin unter anderem Leefmann (2019), Worboys et al. (2021), Woolf (2008). Gemeinsam ist den verschiedenen Definitionsansätzen, dass ein wechselseitiger Erkenntnistransfer zwischen medizinischer Grundlagenforschung und klinischer Forschung im Zentrum der Forschungstätigkeit Translationaler Medizin steht, um die Etablierung von neuen Untersuchungs- und Behandlungsverfahren zu beschleunigen und zu verbessern. Die Verzahnung von medizinischer Grundlagenforschung und klinischer Forschung ist nicht erst für die rezente Medizin von Relevanz, sondern kann für die klinisch-internistische Forschung in der Nachkriegszeit und frühen Bundesrepublik beobachtet werden. Diese These wurde anhand der vorliegenden Fallstudie zur Radioisotopenforschung in der internistischen Klinik diskutiert und soll in weiteren Fallstudien im Rahmen des DFG-Projekts untersucht werden.
